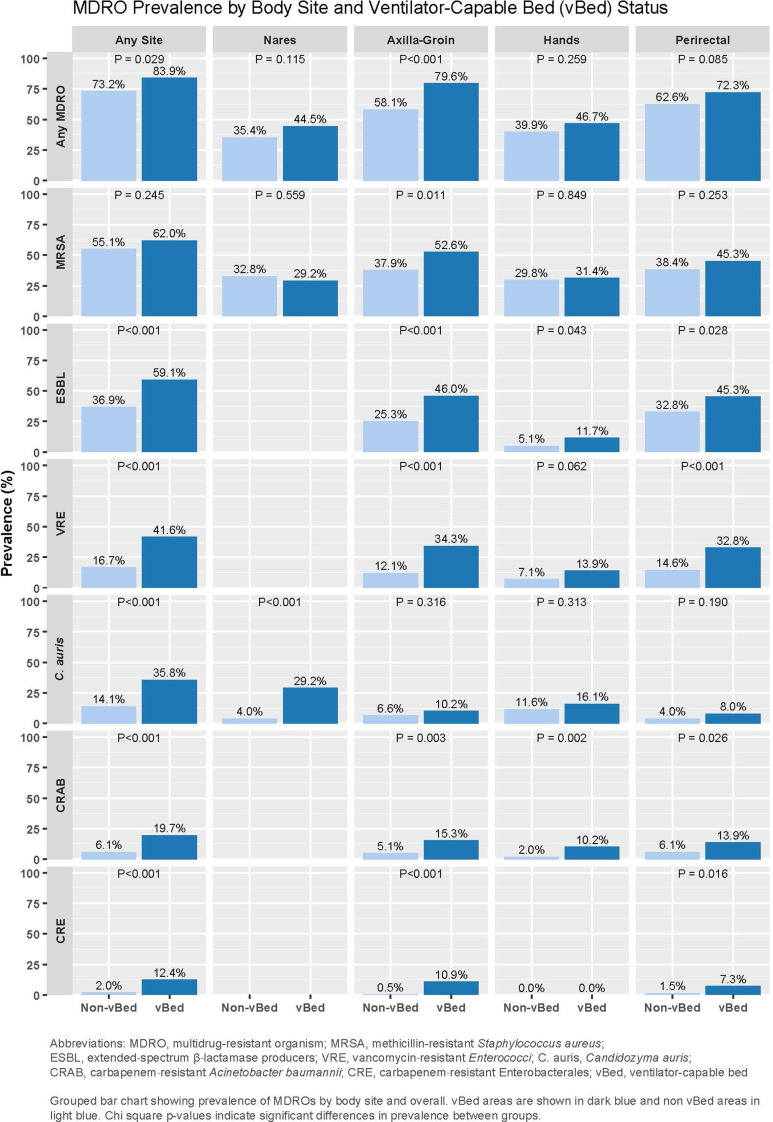# 231 Hospital-Onset Sepsis as a Potential Quality Metric: Risk Adjustment for Facility-Level Benchmarking

**DOI:** 10.1017/ash.2026.10446

**Published:** 2026-06-23

**Authors:** Gabrielle Gussin, Raveena D. Singh, Raheeb Saavedra, Valeria Zaragoza, Jonathan Calzada, Alice Lee, Matthew San Pedro, Jahan Hosseinian, Gabriel Gadia, Julie Shimabukuro, Courtney Myers, Ayesha Khan, Cassiana E. Bittencourt, Susan Huang

**Affiliations:** 1 University of California, Irvine; 2 University of California, Irvine School of Medicine; 3 University of California Irvine School of Medicine; 4 UC Irvine; 5 University of California Irvine Health; 6 UCI

## Abstract

**Background:** vSNFs are known reservoirs of MDROs. However, data are lacking on how MDRO carriage may differ between residents in ventilator-capable (vBed) and non-ventilator-capable (non-vBed) areas within vSNFs. **Methods:** We conducted two point-prevalence MDRO sweeps in two vSNFs (Facility A: 44 vBeds/55 non-vBeds; Facility B: 31 vBeds/167 non-vBeds) from May-June 2025. All occupied beds were sampled except for Facility B’s non-vBeds which randomly sampled 50 beds due to size. Sampling involved hands, axilla/groin and peri-rectal areas cultured for MRSA, ESBL, VRE, C. auris, CRAB, and CRE, plus bilateral nares swabs cultured for MRSA and C. auris. Descriptive statistics summarized overall and MDRO-specific prevalence across body sites, with differences between vBed and non-vBed residents evaluated using t-tests and chi-square tests. **Results:** Overall MDRO prevalence was higher in vBeds (83.9%, 115/137) than non-vBeds (73.2%, 145/198; p=0.029) although the rank order of carriage was similar: MRSA > ESBL > VRE > C. auris > CRAB > CRE. vBed residents had significantly higher prevalence for all pathogens except MRSA (Figure). Multi-MDRO burden was greater in vBeds (mean 2.3 MDROs/resident) than non-vBeds (1.3; p<0.001); vBeds had a substantially higher likelihood of carrying ≥3 (46.0% vs 13.6%; p<0.001), ≥4 (24.1% vs 4.0%; p<0.001), or ≥5 MDROs (10.9% vs 1.0%; p<0.001). Emerging MDROs (CRE, CRAB, C. auris) were more prevalent in vBeds (43.1% vs. 19.7%; p<0.001), and were highly associated with endemic MDRO co-carriage (98.3% [58/59] vBeds; 97.4% [38/39] non?vBeds). The mean number of positive body sites was higher in vBeds (2.4 vs 2.0; p=0.003), with a higher likelihood of being positive at ≥2 sites (73.0% vs 59.6%; p=0.009), ≥3 sites (51.1% vs 39.4%; p=0.017), or 4 sites (35.0% vs 23.7%; p=0.010). vBed residents were more likely to have axilla/groin carriage (79.6% vs 58.1%; p<0.001) with non-significantly higher carriage at other sites (Figure). Nasal carriage patterns differed by organism: MRSA prevalence was similar between vBed and non?vBed residents (29.2% vs 32.8%; p=0.559), whereas C. auris was markedly higher in vBeds (29.2% vs 4.0%; p<0.001). **Conclusion:** Carriage of both endemic and emerging MDROs is widespread in vSNFs, affecting ~75% of residents regardless of ventilator status. However, vBed occupants were more likely to harbor an MDRO, and more likely to harbor multiple MDROs at multiple body sites. Emerging pathogens were also more common in vBed occupants, and were almost always associated with co-carriage of endemic MDROs. Guidance is needed for comprehensive MDRO response in vSNFs.

**Figure:**